# Damage-associated molecular pattern (DAMP) activation in melanoma: investigation of the immunogenic activity of 15-deoxy, Δ^12,14^ prostamide J_2_

**DOI:** 10.18632/oncotarget.27856

**Published:** 2020-12-29

**Authors:** Ahmed Elhassanny, Rene Escobedo, Daniel Ladin, Colin Burns, Rukiyah Van Dross

**Affiliations:** ^1^Department of Pharmacology and Toxicology, Brody School of Medicine, East Carolina University, Greenville, NC, USA; ^2^Medical Doctor Program, Brody School of Medicine, East Carolina University, Greenville, NC, USA; ^3^Department of Chemistry, East Carolina University, Greenville, NC, USA; ^4^Center for Health Disparities, East Carolina University, Greenville, NC, USA

**Keywords:** calreticulin, endoplasmic reticulum stress, dendritic cells, endocannabinoid metabolite

## Abstract

Metastatic melanoma is the most deadly skin neoplasm in the United States. Outcomes for this lethal disease have improved dramatically due to the use of both targeted and immunostimulatory drugs. Immunogenic cell death (ICD) has emerged as another approach for initiating antitumor immunity. ICD is triggered by tumor cells that display damage-associated molecular patterns (DAMPs). These DAMP molecules recruit and activate dendritic cells (DCs) that present tumor-specific antigens to T cells which eliminate neoplastic cells. Interestingly, the expression of DAMP molecules occurs in an endoplasmic reticulum (ER) stress-dependent manner. We have previously shown that ER stress was required for the cytotoxic activity of the endocannabinoid metabolite, 15-deoxy, Δ^12,14^ prostamide J_2_ (15dPMJ_2_). As such, the current study investigates whether 15dPMJ_2_ induces DAMP signaling in melanoma. In B16F10 cells, 15dPMJ_2_ caused a significant increase in the cell surface expression of calreticulin (CRT), the release of ATP and the secretion of high-mobility group box 1 (HMGB1), three molecules that serve as surrogate markers of ICD. 15dPMJ_2_ also stimulated the cell surface expression of the DAMP molecules, heat shock protein 70 (Hsp70) and Hsp90. In addition, the display of CRT and ATP was increased by 15dPMJ_2_ to a greater extent in tumorigenic compared to non-tumorigenic melanocytes. Consistent with this finding, the activation of bone marrow-derived DCs was upregulated in co-cultures with 15dPMJ_2_-treated tumor compared to non-tumor melanocytes. Moreover, 15dPMJ_2_-mediated DAMP exposure and DC activation required the electrophilic cyclopentenone double bond within the structure of 15dPMJ_2_ and the ER stress pathway. These results demonstrate that 15dPMJ_2_ is a tumor-selective inducer of DAMP signaling in melanoma.

## INTRODUCTION

The immune system plays a pivotal role in preventing both the formation and survival of cancer through surveillance and elimination of abnormal cells. Tumor cells circumvent this process using different strategies including the inhibition of cytotoxic [e.g., cytotoxic T cells and natural killer (NK)] and/or the stimulation of immunosuppressive [e.g., regulatory T cells and myeloid derived suppressor cells (MDSC)] cell types [1]. Clinically available immunotherapeutic agents restore the immunosurveillance capabilities of immune cells by disrupting the immunosuppressive interaction between cancer and T cells (e.g., PD-1 and PD-L1) [2]. Based upon the remarkable efficacy of this approach, an intense search is underway to identify other interventions that modulate immunological pathways to prevent tumor survival. A growing body of preclinical evidence and clinical studies indicate that the initiation of immunogenic cell death (ICD) suppresses tumor growth and enhances the activity of existing immunotherapeutics [3–5]. ICD is a type of apoptotic cell death that elicits an adaptive immune response triggered by damage-associated molecular patterns (DAMP). These DAMP molecules are expressed on the surface of or released from tumor cells that are exposed to DAMP inducing agents such as radiotherapy, photodynamic therapy, and a select group of chemotherapeutic agents (e.g., doxorubicin, bortezomib, and oxaliplatin) [5, 6]. It is well-established that an efficacious ICD response requires the display of the DAMPs, calreticulin (CRT), ATP and non-histone chromatin protein high-mobility group box 1 (HMGB1). Once exposed, these molecules bind to cell surface receptors on dendritic cells (DCs) leading to tumor corpse phagocytosis and an increase in DC maturation markers including MHCII, CD80 and CD86. Mature DCs then present tumor-specific antigens to T cells that elicit potent antitumor responses and generate immunological memory [6, 7]. Therefore, agents that induce DAMP-mediated ICD may produce durable therapeutic responses against melanoma and other malignancies.

The endoplasmic reticulum (ER) stress pathway regulates different cellular processes including the emission of DAMP molecules [8, 9]. ER stress occurs when the protein folding load in cells exceeds its folding capacity. To reestablish ER homeostasis, three sensors: PERK, IRE-1, and ATF6 are activated to promote cell survival. However, if the levels of ER stress are insurmountable, transcriptional activation of CHOP10 leads to the initiation of programmed cell death [10]. In numerous studies, DAMP inducing agents required the activity of PERK to initiate DAMP emission and cell death [9, 11]. Moreover, cytotoxic agents that are unable to induce ER stress, such as cisplatin and mitomycin C, were only capable of inducing ICD when co-administered with ER stress inducers such as thapsigargin or tunicamycin [12]. Interestingly, ER stress also plays a role in the selectivity of cytotoxic agents towards tumor cells. ER stress levels are elevated in cancer cells primarily due to the high demand for newly folded proteins in hyperproliferative cells. However, proliferation rates in non-cancerous cells are typically low and as a consequence, these cells have minimal protein folding loads and ER stress levels. Hence, ER stress inducing agents cause death preferentially in cancer cells because the death threshold is more readily reached [10, 13]. This selective tumor targeting often results in reduced adverse effects, a desired property for chemotherapeutic agents.

15-deoxy, Δ^12,14^ prostamide J_2_ (15dPMJ_2_) is a metabolic product of the endocannabinoid, arachidonoyl ethanolamide (AEA). 15dPMJ_2_ suppresses the growth of B16F10 melanoma tumors in C57BL/6 mice [13]. 15dPMJ_2_ is also cytotoxic towards non-melanoma skin cancer (NMSC) and colon cancer cell lines as well as primary patient melanoma cells. In addition, 15dPMJ_2_ induced cell death is mediated by the ER stress pathway. Hence, the goal of the current study was to determine whether 15dPMJ_2_ activates DAMPs in melanoma cells. Moreover, the role of ER stress in 15dPMJ_2_-induced DAMP signaling was explored. Our data demonstrate that 15dPMJ_2_ causes tumor-directed DAMP exposure and DC activation through a process that relies on the ER stress pathway.

## RESULTS

### 15dPMJ_2_ induces DAMP exposure in tumor cells

**Figure 1:**
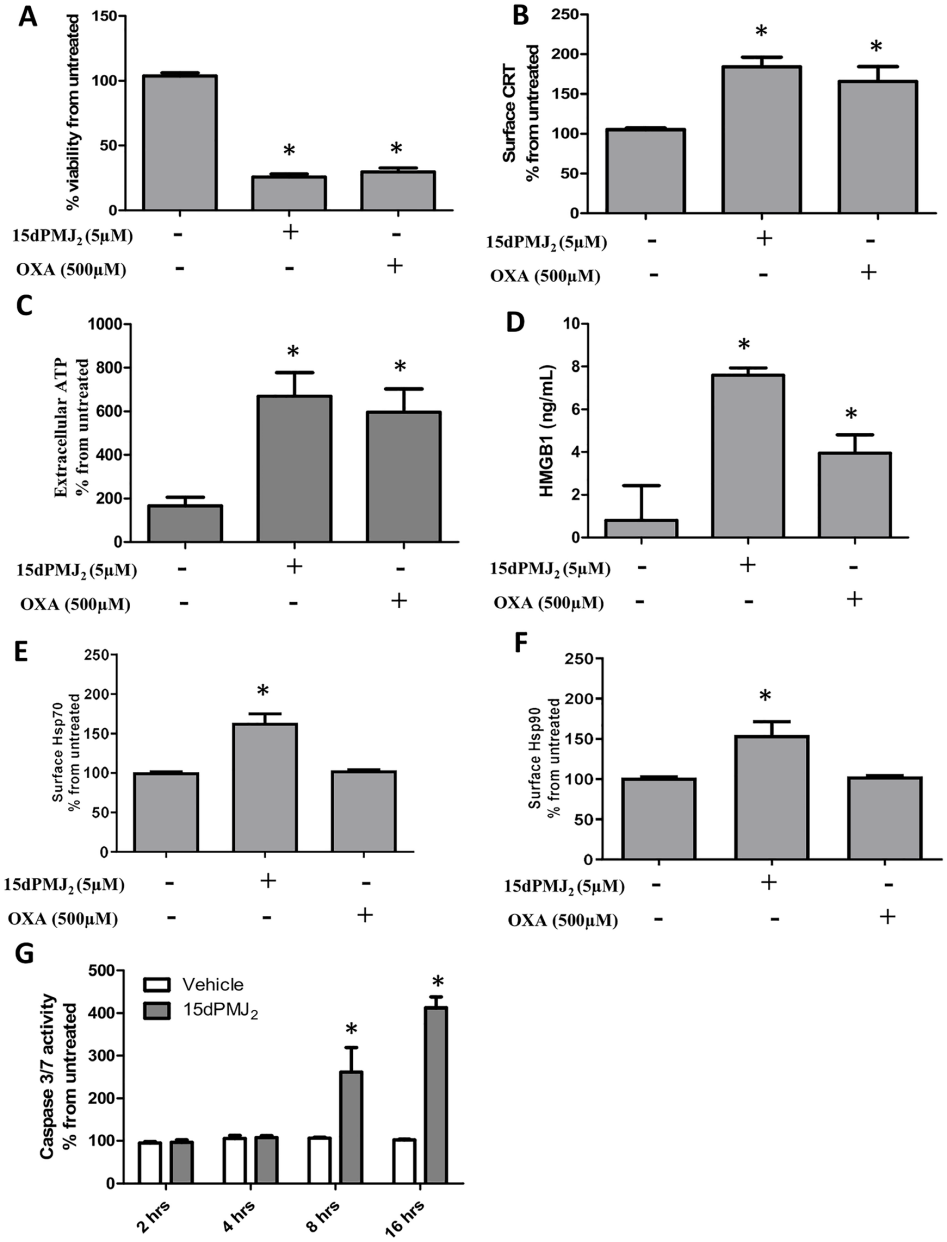
15dPMJ_2_ increases DAMP expression and cell death in B16F10 melanoma cells. (**A**–**F**) B16F10 cells were treated with vehicle (0.1% DMSO), 5 μM 15dPMJ_2,_ 500 μM oxaliplatin, or the cells were left untreated. (A) Cell viability was evaluated by performing MTS assays after 24 hours of treatment. (B) The cell surface expression of calreticulin (CRT) was measured by conducting flow cytometric analysis after treating the cells for 2 hours with the indicated agents. (C) Extracellular ATP levels were measured using the CellTiter-Glo 2.0 assay kit after treating the cells for 4 hours. (D) Extracellular HMGB1 was measured by using the HMGB1 ELISA kit after cell treatment for 24 hours. (E and F) The cell surface expression of Hsp70 (E) and Hsp90 (F) was measured by conducting flow cytometric analysis after treating the cells for 4 hours. (**G**) B16F10 cells were treated with 5 μM 15dPMJ_2_ or vehicle for 2, 4, 8, or 16 hours. Caspase-3/7 activity was measured by using Caspase-Glo 3/7 reagent as directed in the manufacturer’s instructions. Sample values in B, C, E and F are displayed as the percentage from untreated cells (% from untreated). The data were analyzed using one-way ANOVA followed by Tukey’s multiple comparison test and are represented as the mean ± SEM of three independent experiments. ^*^ *p <* 0.05, sample compared to vehicle-treated cells.

To verify the cytotoxicity of 15dPMJ_2_, B16F10 melanoma cells were treated with 15dPMJ_2_ and cell survival was examined. As a positive control, the cells were also treated with the prototype DAMP-ICD inducer, oxaliplatin. 15dPMJ_2_ and oxaliplatin significantly reduced the viability of B16F10 cells with similar levels of death occurring at a substantially lower concentration of 15dPMJ_2_ (5 μM) than oxaliplatin (500 μM) (Figure 1A). We then examined whether 15dPMJ_2_ stimulated the exposure of the surrogate ICD markers; CRT, ATP, and HMGB1 [14]. Comparable levels of cell surface CRT and extracellular ATP were detected in cells treated with 15dPMJ_2_ and oxaliplatin (Figure 1B and 1C). However, the release of HMGB1 was substantially greater in 15dPMJ_2_ compared to oxaliplatin treated cells (Figure 1D). It has been demonstrated that agents that trigger the exposure of a wide variety of DAMPs are efficient inducers of ICD [15, 16]. As such, the exposure of the DAMPs, Hsp70 and Hsp90, was also examined. Hsp70 and Hsp90 were upregulated on the surface of B16F10 cells that were treated with 15dPMJ_2_ but not oxaliplatin (Figure 1E and 1F). The exposure of DAMPs induced by 15dPMJ_2_ was investigated in other cancer cell types. 15dPMJ_2_ also increased the cell surface expression of CRT in human non-melanoma skin cancer (A431) and colorectal cancer (HT29) cell lines (Supplementary Figure 1A and 1B). Several studies have revealed that the exposure of CRT and ATP occurs prior to apoptosis rather than as a consequence of apoptosis [9]. Hence, the sequence of 15dPMJ_2_-induced DAMP exposure was investigated. Caspase 3/7 activity and phosphatidylserine exposure, two indicators of apoptosis, were initially detected after 8 hours of treatment with 15dPMJ_2_ (Figure 1G and Supplementary Figure 2), whereas the display of CRT and ATP occurred after 2 and 4 hours, respectively. This indicates that the exposure of CRT and ATP induced by 15dPMJ_2_ were pre-apoptotic events similar to other DAMP-ICD inducing agents [17].

### 15dPMJ_2_ increases DAMP display preferentially in tumor cells

**Figure 2:**
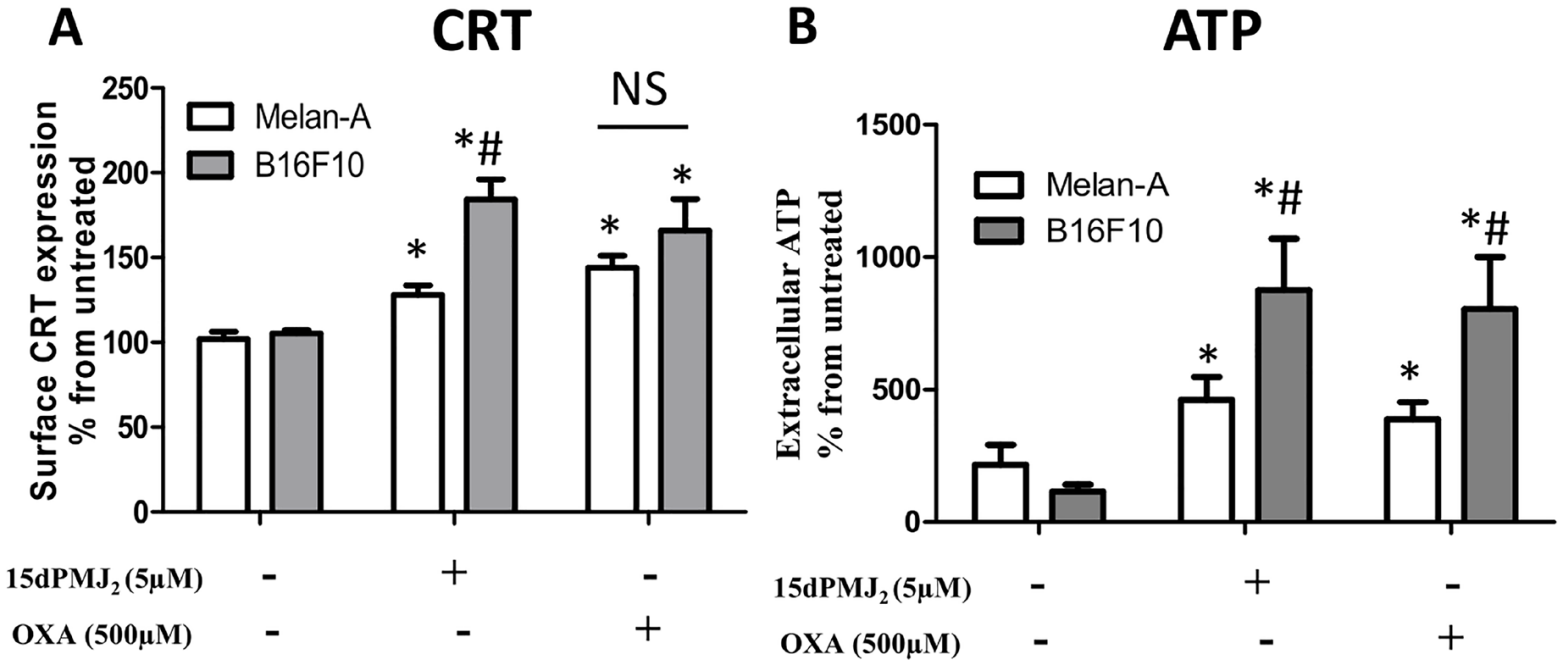
15dPMJ_2_ increases DAMP expression selectively in melanoma cells. (**A** and **B**) Tumorigenic (B16F10) and non-tumorigenic (Melan-A) melanocytes were treated with vehicle (0.1% DMSO), 5 μM 15dPMJ_2,_ 500 μM oxaliplatin or the cells remained untreated. (A) The cell surface expression of calreticulin (CRT) was measured by conducting flow cytometric analysis after cell treatment for 2 hours. (B) The extracellular levels of ATP were detected after 4 hours of treatment by using CellTiter-Glo^®^ kit. Sample values are displayed as the percentage from untreated cells (% from untreated). The data were analyzed using one-way ANOVA followed by Tukey’s multiple comparison test and are represented as the mean ± SEM of three independent experiments. ^*^ *p <* 0.05, sample compared to vehicle treated cells; ^#^ *p <* 0.05, sample compared to Melan-A cells; NS, not statistically different.

Our previous study showed that 15dPMJ_2_ exhibited greater cytotoxicity towards tumorigenic than non-tumorigenic melanocytes [13]. We found that this tumor-selective death was regulated by the ER stress pathway. Since it has been reported that ER stress is necessary for CRT and ATP exposure [9, 18], we investigated whether 15dPMJ_2_-induced DAMP expression occurred preferentially in tumors. To examine tumor-selective DAMP induction, we utilized the B16F10 (tumorigenic) and Melan-A (non-tumorigenic) cell lines that were derived from C57BL/6 mice. In the presence of 15dPMJ_2_, the display of CRT and ATP was significantly elevated in tumorigenic compared to non-tumorigenic melanocytes (Figure 2A and 2B). In oxaliplatin-treated cells ATP, instead of CRT, was preferentially induced in tumor cells (Figure 2A and 2B). We also investigated the selectivity of 15dPMJ_2_ in keratinocytes. Similar to our observation in melanocytes, 15dPMJ_2_ stimulated cell surface CRT expression in tumorigenic (A431 cells) as opposed to non-tumorigenic (HaCaT cells) keratinocytes (Supplementary Figure 1C). Hence, 15dPMJ_2_ causes the exposure of key DAMPs selectively in tumor cells.

### 15dPMJ_2_-induced DAMP exposure activates dendritic cells

**Figure 3:**
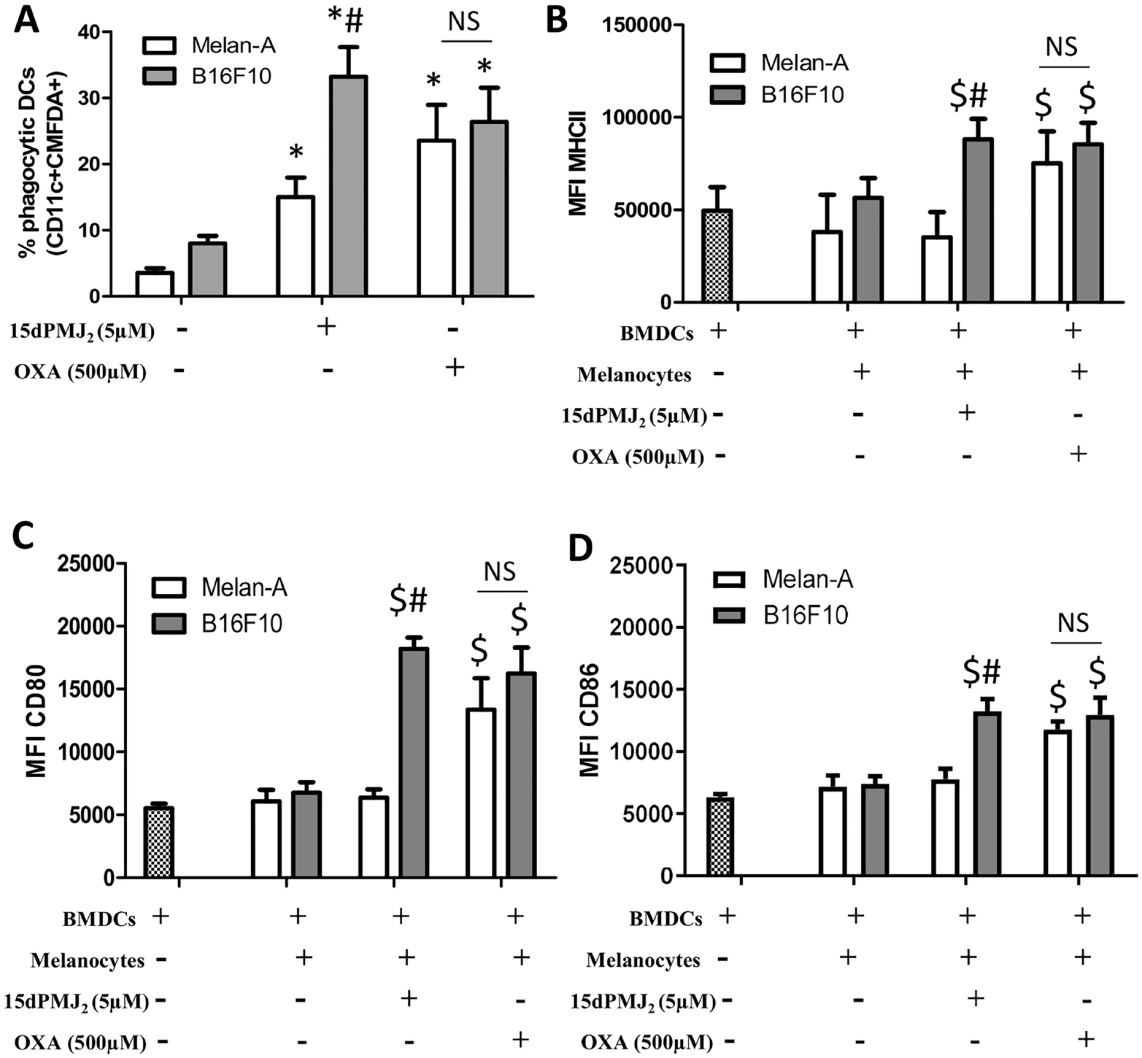
15dPMJ_2_-treated tumorigenic melanocytes increased the phagocytotic activity and maturation of DCs. (**A**) B16F10 and Melan-A cells were prelabeled with CellTracker Green-CMFDA and then treated for 24 hours with vehicle (0.1% DMSO), 5 μM 15dPMJ_2_ or 500 μM oxaliplatin. Bone marrow-derived DCs were differentiated for 8 days and then co-incubated with the labeled melanocytes for 2 hours. The phagocytic activity of the DCs was measured by conducting flow cytometric analysis to identify CD11c+/CMFDA+ cells. (**B**–**D**) Differentiated DCs were cultured without melanocytes or co-cultured for 24 hours with B16F10 or Melan-A cells that were treated with vehicle (0.1% DMSO), 5 μM 15dPMJ_2_, or 500 μM oxaliplatin. The expression of (B) MHCII, (C) CD80 and (D) CD86 on the surface of DCs was measured by conducting flow cytometry analysis. The data were analyzed using one-way ANOVA followed by Tukey’s multiple comparison test and are represented as the mean fluorescence intensity (MFI) ± SEM of three independent experiments. ^*^ *p <* 0.05, sample compared to DCs co-cultured with vehicle-treated melanocytes; ^#^ *p <* 0.05, B16F10 sample compared to treatment-matched Melan-A cells; ^$^ *p <* 0.05, sample compared to DCs cells cultured without melanocytes; NS, not statistically different.

**Figure 4:**
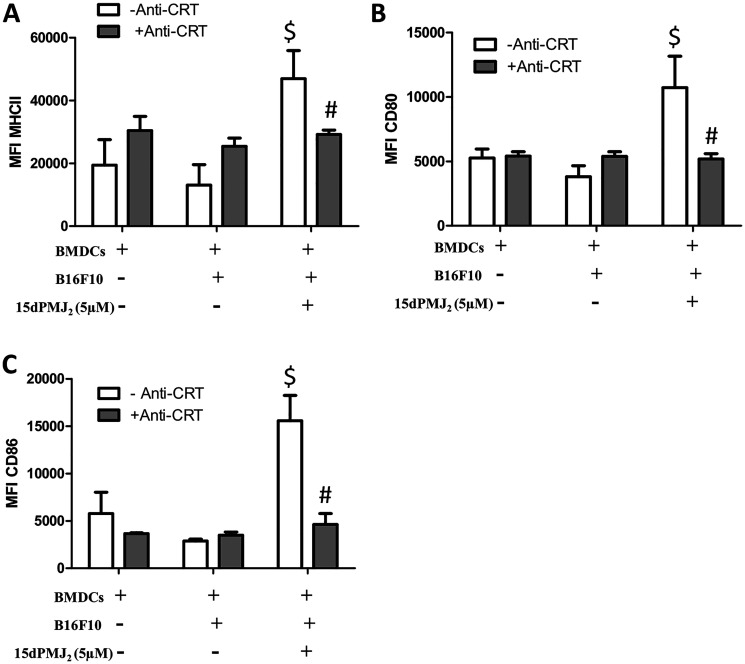
Cell surface calreticulin expression is required for 15dPMJ_2_-mediated DC activation. (**A**–**C**) B16F10 cells were pretreated with or without calreticulin blocking antibody (anti-CRT) and then treated with vehicle (0.1% DMSO) or 5 μM 15dPMJ_2_. Differentiated DCs were then cultured with or without the B16F10 cells for 24 hours. The expression of (A) MHCII, (B) CD80, and (C) CD86 on the surface of the DCs was measured by conducting flow cytometry analysis. The data were analyzed using one-way ANOVA followed by Tukey’s multiple comparison test and are represented as the mean fluorescence intensity (MFI) ± SEM of three independent experiments. ^$^ *p <* 0.05, sample compared to DCs cells cultured without melanocytes. ^#^ *p <* 0.05, sample compared to 15dPMJ_2_-treated co-cultures without anti-CRT.

Tumor-generated DAMPs bind to cell surface receptors on DCs to increase its phagocytotic activity. DCs then increase the expression of maturation markers including, MHCII, CD80 and CD86 [15, 19]. Therefore, we investigated whether 15dPMJ_2_-induced DAMP exposure leads to DC activation. To evaluate DC phagocytic activity, B16F10 and Melan-A cells were labeled with the tracking dye, CMFDA, and the cells were treated with 15dPMJ_2_, oxaliplatin or vehicle. The labeled melanocytes were then co-incubated with naïve, bone marrow-derived DCs that were extracted from C57BL/6 mice. Treatment of B16F10 cells with 15dPMJ_2_ or oxaliplatin stimulated its phagocytosis by DCs (Figure 3A). Also, a substantially greater percentage of DCs engulfed 15dPMJ_2_-treated B16F10 than Melan-A cells (Figure 3A). However, comparable levels of phagocytotic activity were observed in the presence of oxaliplatin-treated B16F10 and Melan-A cells, indicating an absence of selectivity (Figure 3A). Next, we examined the effect of DAMP expression on the elaboration of DC maturation markers. 15dPMJ_2_-treated B16F10 but not Melan-A cells increased the expression of MHCII, CD80, and CD86 on the surface of DCs (Figure 3B–3D). In contrast to this observation, oxaliplatin-treated B16F10 and Melan-A cells caused comparable levels of DC maturation, consistent with the DC phagocytotic activity (Figure 3A–3D). Of note, similar levels of DC activity were stimulated by 15dPMJ_2_ and oxaliplatin but with a substantially lower concentration of 15dPMJ_2_ (Figure 3). Next, to confirm that 15dPMJ_2_-induced DC maturation was driven by DAMPs, a CRT blocking antibody was utilized. Blockade of cell surface CRT in 15dPMJ_2_-treated B16F10 cells suppressed the exposure of MHCII, CD80 and CD86 on DCs (Figure 4). These collective results demonstrate that 15dPMJ_2_ is a tumor-selective DAMP and DC stimulant, a feature that is not displayed by the prototype DAMP inducer, oxaliplatin.

### ER stress is required for 15dPMJ_2_-induced DAMP signaling in B16F10 cells

**Figure 5:**
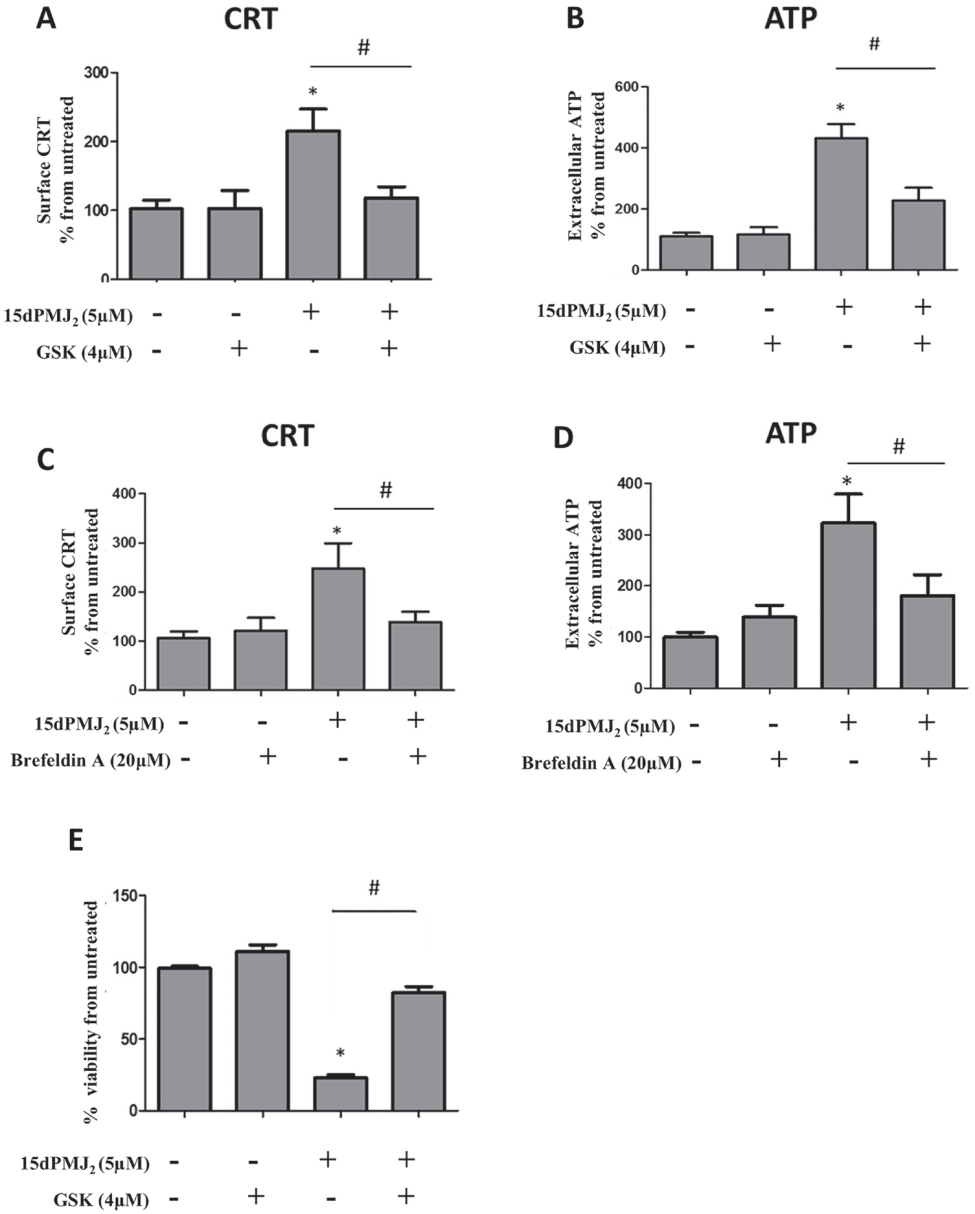
ER stress is required for 15dPMJ_2_-induced DAMP expression. (**A** and **B**) B16F10 cells were pre-treated for 1 hour with 4 μM GSK2606414 (GSK) or vehicle (0.1% DMSO) and then treated with 5 μM 15dPMJ_2_ or vehicle. (A) Cell surface calreticulin (CRT) was measured by conducting flow cytometry analysis on cells that were treated for 2 hours. (B) Extracellular ATP levels were measured after 4 hours using the CellTiter-Glo 2.0 assay kit. (**C** and **D**) B16F10 cells were pre-treated with 20 μM Brefeldin A or vehicle (0.1% DMSO) for 1 hour and then treated with 5 μM 15dPMJ_2_ or vehicle. (C) Cell surface CRT expression was measured by performing flow cytometric analysis after 2 hours of treatment. (D) Extracellular ATP was measured in cells that were treated for 4 hours. (**E**) B16F10 cells were pre-treated for 1 hour with 4 mM GSK or vehicle and then treated with 5 μM 15dPMJ_2_ or vehicle. Cell viability was evaluated by conducting MTS assays after 24 hours of treatment. Sample values are displayed as the percentage viability from untreated cells (% viability from untreated). The data represent the mean ± SEM of three independent experiments. ^*^ *p* < 0.05, sample compared to vehicle-treated cells; ^#^ *p* < 0.05, sample compared to 15dPMJ_2_.

**Figure 6:**
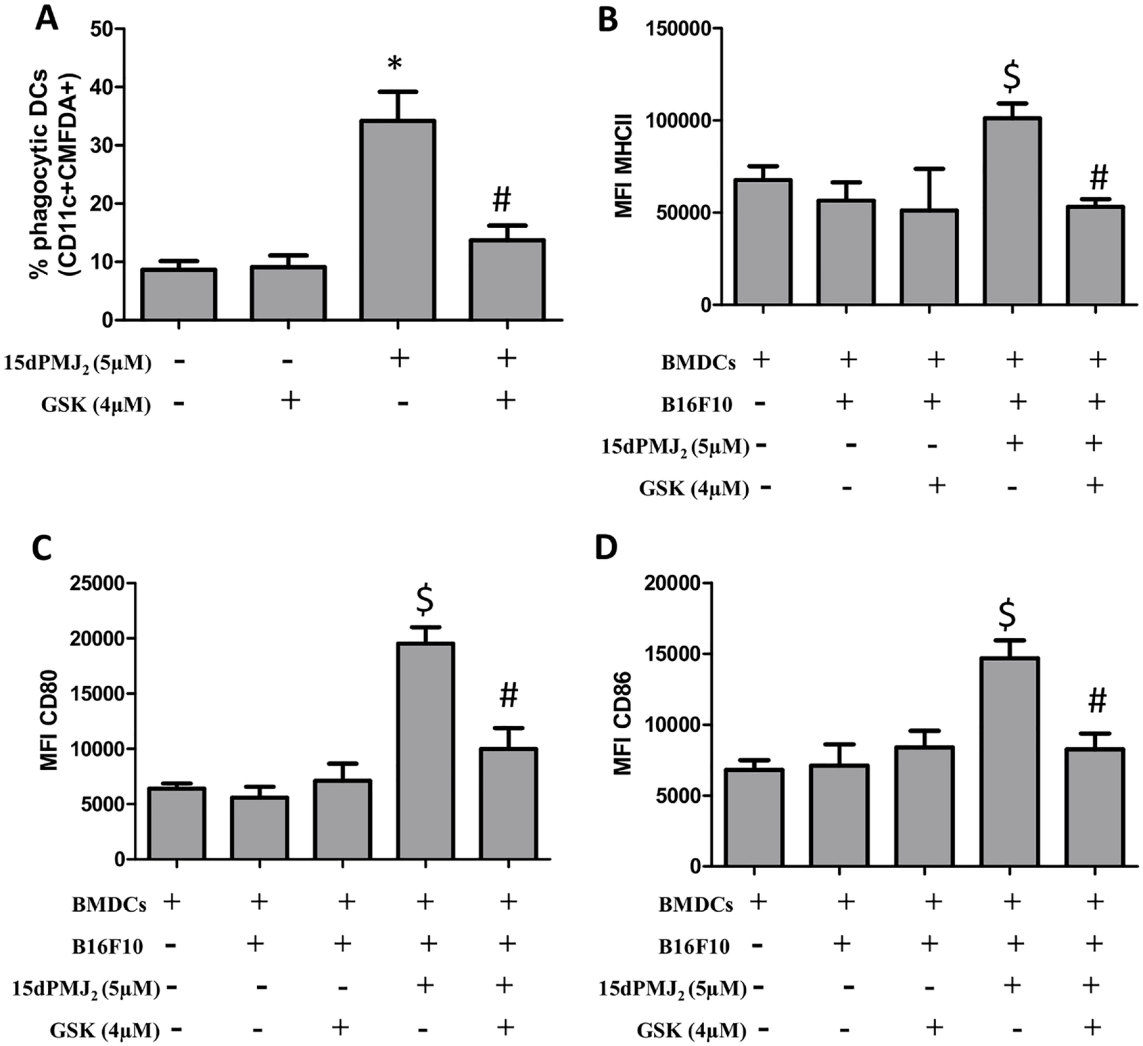
ER stress is essential for DC activation by 15dPMJ_2_-treated tumorigenic melanocytes. (**A**) B16F10 cells were labeled with CellTracker Green-CMFDA, pretreated with 4 μM GSK2606414 (GSK) or vehicle (0.1%DMSO) for 1 hour, and then treated with vehicle or 5 μM 15dPMJ_2_ for 24 hours. Differentiated DCs were co-incubated with the B16F10 cells for 2 hours. Phagocytic DCs were detected as CD11c+CMFDA+ cells. (**B**–**D**) Differentiated DCs were cultured without melanocytes or co-cultured with B16F10 cells. The B16F10 cells were pretreated with 4 μM GSK for 1 hour and then treated with vehicle or 5 μM 15dPMJ_2_ for 24 hours. The expression of (B) MHCII, (C) CD80 and (D) CD86 on the surface of DCs was detected by conducting flow cytometric analysis. The data were analyzed using one-way ANOVA followed by Tukey’s multiple comparison test and values are represented as the mean fluorescence intensity (MFI) ± SEM of three independent experiments. ^*^ *p <* 0.05, sample compared to DCs co-cultured with vehicle-treated B16F10 cells; ^#^ *p <* 0.05, sample compared to 15dPMJ_2_-treated co-cultures; ^$^ *p <* 0.05, sample compared to DCs cells cultured without melanocytes.

The display of CRT and the release of ATP are regulated by the ER stress pathway [9, 18]. In different model systems, PERK was the predominant regulator of DAMP induction and cell death [9, 11]. Therefore, to gain insight into mechanisms underlying 15dPMJ_2_-induced DAMP exposure, the ER stress pathway was probed utilizing the small-molecule PERK inhibitor, GSK2606414 [10]. 15dPMJ_2_-mediated cell surface expression of CRT was abrogated by GSK2606414 in B16F10 cells (Figure 5A). In addition, blockade of PERK with the inhibitor prevented the release of ATP (Figure 5B). To determine whether CRT and ATP were exposed via the classical secretory pathway as demonstrated in other studies, anterograde protein transport from the ER to the Golgi apparatus was blocked by using Brefeldin A [9]. Obstructing the secretory pathway inhibited 15dPMJ_2_-induced CRT display and ATP release (Figure 5C and 5D). Furthermore, the cytotoxicity of 15dPMJ_2_ was suppressed by GSK2606414 demonstrating the need for ER stress in DAMP expression and death (Figure 5E; [13]). Next, to examine the impact of the ER stress pathway on DC activation, B16F10 cells were pretreated with GSK2606414, treated with 15dPMJ_2_ and then co-cultured with DCs (Figure 6). The engulfment of 15dPMJ_2_-treated B16F10 cells by DCs was prevented by inhibiting ER stress (Figure 6A). Similarly, the upregulation of MHCII, CD80, CD86 on DCs was abrogated when the ER stress response was blunted (Figure 6B–6D). Hence, both the ER stress and classical secretory pathways are employed by 15dPMJ_2_ to elicit DAMP expression and DC activation.

### The electrophilic α,β-unsaturated carbonyl group in 15dPMJ_2_ regulates the exposure of DAMPs

**Figure 7:**
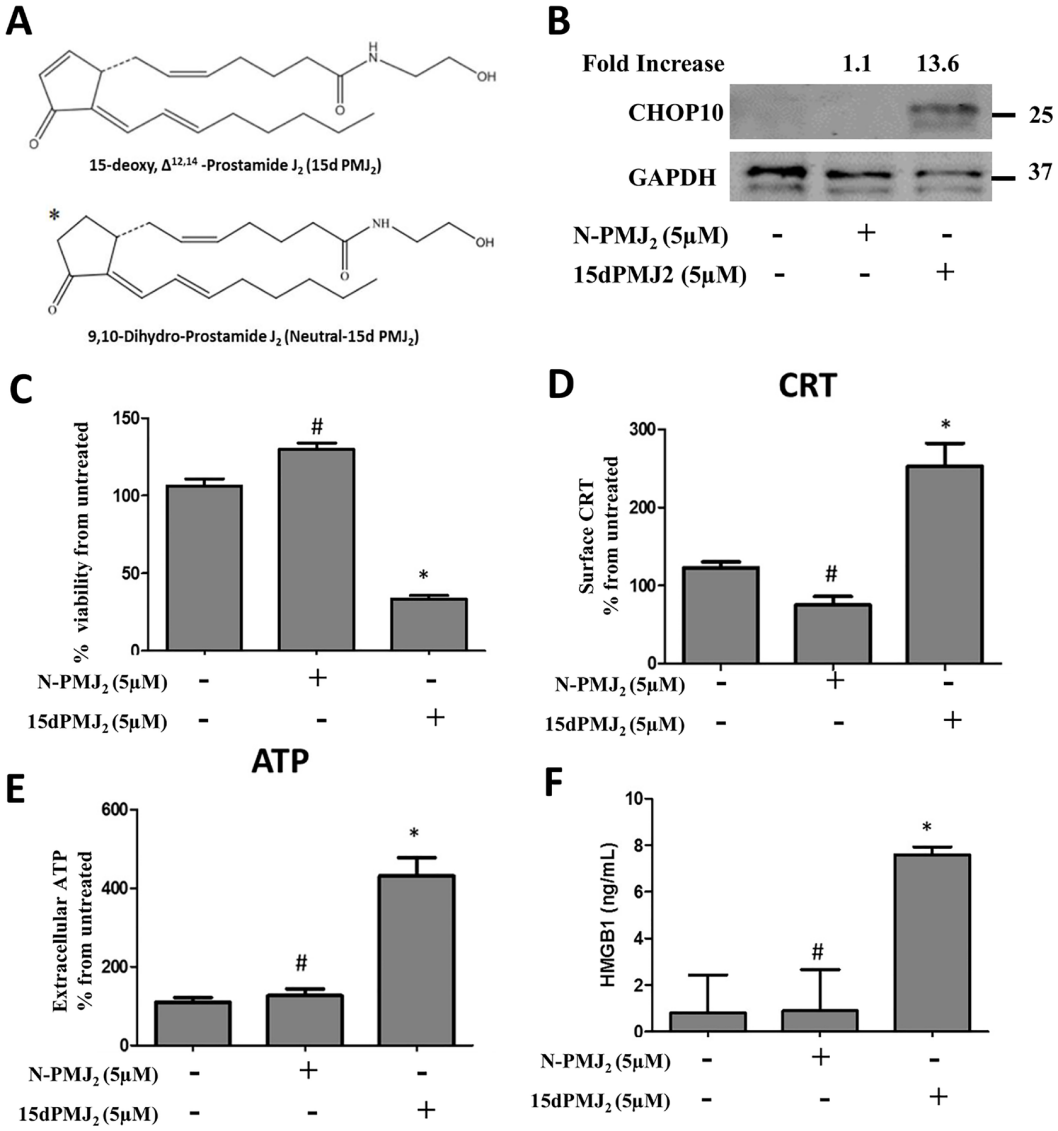
The α,β-unsaturated carbonyl moiety in 15dPMJ_2_ regulates DAMP activation. (**A**) Chemical structures of 15dPMJ_2_ and neutral-15dPMJ_2_ (N-PMJ_2_). (**B**) B16F10 cells were treated with 5 μM N-PMJ_2_, 5 μM 15dPMJ_2,_ or vehicle (0.1% DMSO) for 6 hours. The levels of CHOP10 protein expression were examined by conducting Western blot analysis. Band intensities were quantified using ImageJ software. The fold increase in protein levels was determined by comparing the band intensity of samples to vehicle-treated cells after normalizing the values to GAPDH levels. (**C**–**F**) B16F10 cells were treated with 5 μM N-PMJ_2_, 5 μM 15dPMJ_2_, or vehicle. (C) Cell viability was examined by conducting MTS experiments after 24 hours of treatment. (D) The cell surface expression of CRT was measured by performing flow cytometric analysis after 2 hours of treatment. (E) Extracellular ATP levels were measured by using the CellTiter-Glo 2.0 kit after 4 hours of treatment. (F) Extracellular HMGB1 was measured by using HMGB1 ELISA kits after 24 hours of treatment. (C–E) Sample values are displayed as the percentage from untreated cells (% from untreated). Data represent the mean ± SEM of three independent experiments. ^*^ *p* < 0.05, sample compared to vehicle-treated cells; ^#^ *p* < 0.05, sample compared to 15dPMJ_2_.

**Figure 8:**
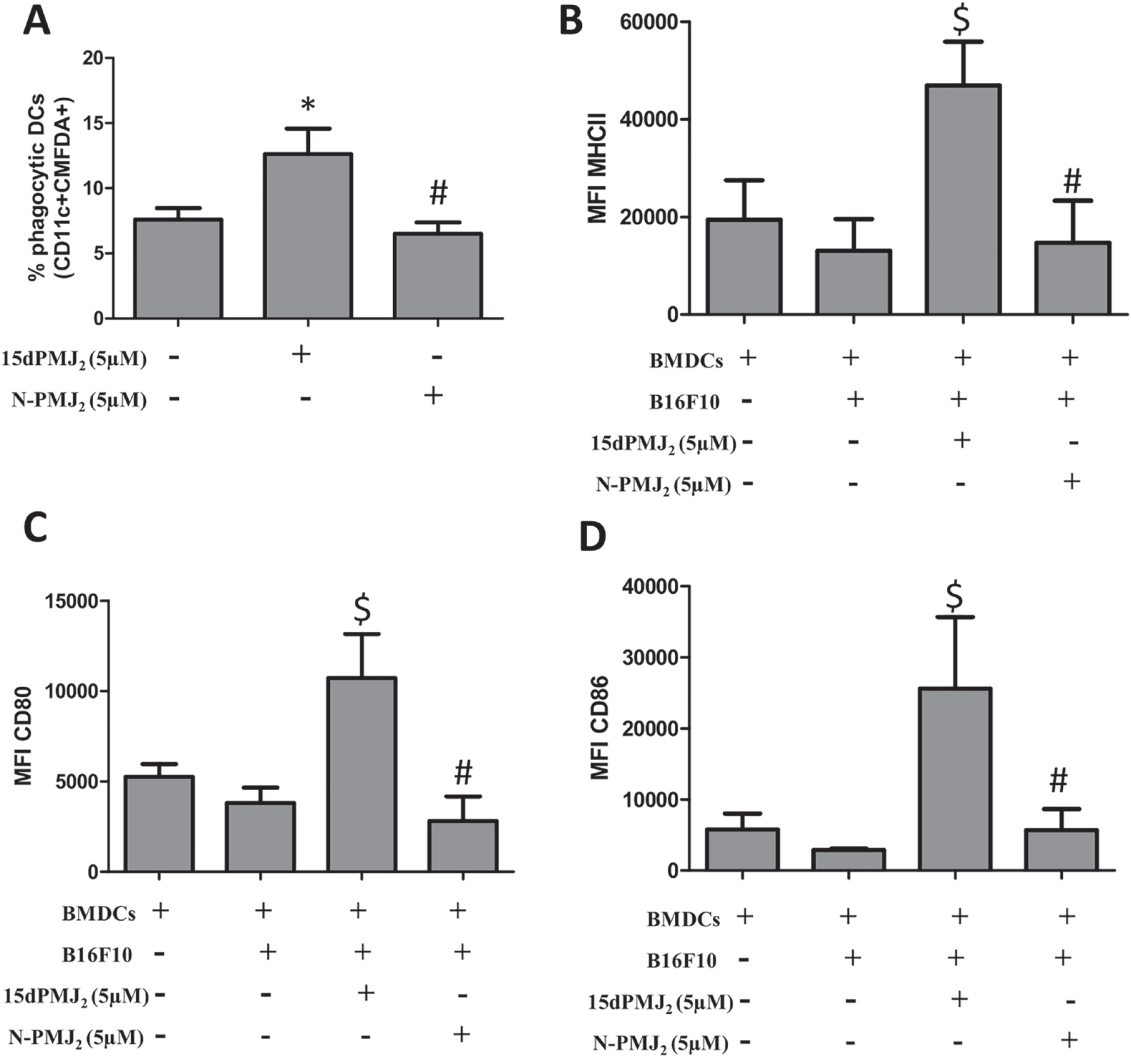
The α,β-unsaturated carbonyl moiety in 15dPMJ_2_ is required for DC activation by 15dPMJ_2_-treated tumorigenic melanocytes. (**A**) B16F10 cells were prelabeled with CellTracker Green-CMFDA and then treated with vehicle (0.1%DMSO), 5 μM 15dPMJ_2_ or 5 μM neutral-15dPMJ_2_ (N-PMJ_2_) for 24 hours. Differentiated DCs were co-incubated with the B16F10 cells for 2 hours. Phagocytic DCs were detected as CD11c+CMFDA+ cells. (**B**–**D**) DCs were co-cultured for 24 hours with B16F10 cells that were treated with vehicle, 5 μM 15dPMJ_2_ or 5 μM N-PMJ_2_ or the DCs were cultured without B16F10 cells. The expression of (B) MHCII, (C) CD80 and (D) CD86 on the surface of DCs was detected by conducting flow cytometric analysis. The data were analyzed using one-way ANOVA followed by Tukey’s multiple comparison test and values are represented as the mean fluorescence intensity (MFI) ± SEM of three independent experiments. ^*^ *p <* 0.05, sample compared to DCs co-cultured with vehicle-treated B16F10 cells; ^#^ *p <* 0.05, sample compared to 15dPMJ_2_-treated co-cultures; ^$^ *p <* 0.05, sample compared to DCs cells cultured without melanocytes.

The current study demonstrates that ER stress is required for 15dPMJ_2_-induced DAMP expression, DC activation and tumor death. Previous studies by our group showed that ER stress and cell death were mediated by the reactive α,β-unsaturated carbonyl group in the cyclopentenone ring of 15dPMJ_2_ [13]. Therefore, the impact of this reactive moiety on DAMP signaling was explored utilizing neutral-15dPMJ_2_, a structural analog of 15dPMJ_2_ which lacks its cyclopentenone double bond (Figure 7A). In B16F10 cells, 15dPMJ_2_ but not neutral-15dPMJ_2_ increased the expression of the cytotoxic ER stress marker, CHOP10 (Figure 7B). Also, 15dPMJ_2_, instead of neutral-15dPMJ_2_ was capable of inducing cell death (Figure 7C). Furthermore, the cell surface expression of CRT, the release of ATP and the secretion of HMGB1 occurred in the presence of 15dPMJ_2_ but not the neutral analog (Figure 7D–7F). Moreover, the phagocytic activity and maturation of DCs were induced by 15dPMJ_2_ but not neutral-15dPMJ_2_ (Figure 8). These data establish that the reactive double bond in 15dPMJ_2_ is required for the expression of DAMPs and the subsequent activation of DCs.

## DISCUSSION

DAMP inducers have emerged as agents that can enhance antigen presenting cell function, stimulate T cell responses, and promote tumor death [3, 20]. These inducers depend on ER stress to increase the exposure/release of DAMP molecules which transform immunologically silent tumor cells into entities that provoke an immune response [8, 21]. According to our previous studies, the *in vitro* and *in vivo* cytotoxicity of the endocannabinoid metabolite, 15dPMJ_2_ is driven by the ER stress pathway [13]. Hence, the current study investigated the ability of 15dPMJ_2_ to stimulate DAMP-mediated immunogenicity in melanoma. We determined that 15dPMJ_2_ significantly increased DAMP expression in different tumor cell types. In addition, DAMP expression was preferentially upregulated in tumorigenic compared to non-tumorigenic cells that were treated with 15dPMJ_2_. Melanoma cells that were treated with 15dPMJ_2_ also caused DC activation and maturation. Moreover, ER stress and the cyclopentenone double bond in 15dPMJ_2_ were identified as critical determinants of 15dPMJ_2_-mediated DAMP signaling. These collective findings suggest that 15dPMJ_2_ may possess immunotherapeutic efficacy against cancer.

A goal of the current investigation was to determine if 15dPMJ_2_ was a genuine DAMP-ICD inducer. Therefore, we examined well-established properties of DAMP signaling including the exposure of the surrogate ICD markers: CRT, ATP, and HMGB1 [14]. The display of these three markers along with the induction of cell death is essential for generating an immune response. Our data showed that 15dPMJ_2_ caused the cell surface expression of CRT, the release of ATP, the secretion of HMGB1 and apoptotic cell death (Figure 1). Furthermore, 15dPMJ_2_-mediated exposure of CRT and ATP required ER stress and the classical secretory pathway, two well-known properties of DAMP induction [9, 18]. Also, 15dPMJ_2_-induced CRT and ATP exposure occurred prior to the cell surface display of phosphatidylserine, while HMGB1 was released passively from the apoptotic cells [5, 9]. Collectively, this suggests that the small molecule prostamide, 15dPMJ_2_, is a bona-fide DAMP inducer.

Numerous studies, conducted primarily by the Kroemer and Zitvogel groups, have determined that DAMPs activate DCs which then initiate a cascade of events that culminate in cytotoxic T cell activation, memory T cell expansion and tumor resolution [17, 22–25]. Hence, DAMP molecules play a decisive role in stimulating DCs which, in turn, trigger the ICD response. However, the manner in which DAMPs are exposed dictates whether these molecules stimulate or inhibit DC activation. For instance, cell surface CRT serves as an “eat me” signal that promotes phagocytosis of the cell by DCs however, secreted CRT inhibits DC-mediated tumor phagocytosis and promotes immunosuppression [5, 26]. Extracellular ATP recruits myeloid precursors that differentiate into mature DCs with antigen presenting activity but adenosine, the metabolic product of ATP, is a potent immunosuppressant that inhibits the antitumor activity of DCs and increases the activity of pro-tumorigenic MDSCs [27–29]. In addition, the reduced form of secreted HMGB1 promotes the processing and presentation of tumor-associated antigens by DCs, however, the fully oxidized HMGB1 is inactive and unable to promote DC activation [30–32]. Since DAMP molecules can either stimulate or inhibit DCs, we tested the consequences of 15dPMJ_2_-mediated DAMP expression on DC function. According to our data, 15dPMJ_2_-treated B16F10 cells were engulfed by DCs that were derived from C57BL/6 mice. Furthermore, the co-culture of DCs with 15dPMJ_2_-treated B16F10 cells increased DC maturation. In addition, blockade of cell surface CRT inhibited the activation of DCs by 15dPMJ_2_-treated B16F10 cells. Since our previous data demonstrated that 15dPMJ_2_ also inhibited subcutaneous B16F10 tumor growth in immunocompetent C57BL/6 mice [13], 15dPMJ_2_ likely activates DCs and this immune cell type may drive the antitumor activity of 15dPMJ_2_. As such, studies are being conducted by our group to investigate 15dPMJ_2_-mediated DAMP-ICD *in vivo*.

Melanoma survival has been dramatically improved due to the use of immune checkpoint inhibitors (ICIs), however, in numerous patients, the associated immune-related adverse effects (irAE) limit their utility [33]. ICIs disturb the immunosuppressive interaction between checkpoint proteins on T cells (e.g., PD-1) and their ligands on tumor cells (e.g., PD-L1) thereby unleashing T cell-mediated cytotoxicity towards the tumor [34]. Because non-tumorigenic cells also express checkpoint ligands, ICIs release cytotoxic activity towards non-tumor tissues and can cause life-threatening irAE. Therefore, an important goal of this study was to determine whether 15dPMJ_2_-stimulated DAMP signaling was selectively initiated in tumor cells. The current study shows that 15dPMJ_2_ preferentially increased both CRT and ATP exposure in tumorigenic compared to non-tumorigenic cells (Figure 2). Moreover, the phagocytotic activity and maturation of DCs was seen in the presence of tumorigenic instead of non-tumorigenic cells treated with 15dPMJ_2_. On the other hand, oxaliplatin failed to cause tumor-selective CRT display or DC activity. The absence of tumor-directed action by oxaliplatin and other non-selective DAMP inducers may generate immune-related adverse effects similar to the immunotherapeutic checkpoint inhibitors. Hence, our finding that 15dPMJ_2_ induces tumor-selective immunogenicity is significant because it implies that the ensuing T cell response will be directed towards the tumor, thereby producing limited irAE.

According to the literature, ER stress plays a crucial role in DAMP-ICD [8, 9]. A report from Panaretakis et al., showed that the downregulation of PERK prevented anthracycline-induced CRT exposure and diminished ICD *in vivo* [11]. Garg and colleagues found that the depletion of PERK decreased hypericin-photodynamic therapy (Hyp-PDT)-mediated CRT display and ATP secretion [9]. In alignment with these results, we determined that the blockade of PERK with GSK2606414, suppressed 15dPMJ_2_-induced CRT exposure, ATP secretion, and DC function. In addition, the neutral analog of 15dPMJ_2_, which is devoid of ER stress inducing activity [13], was unable to elicit CRT or ATP exposure. We also found that the suppression of PERK (Figure 5E) or the enhancement of protein chaperone activity abolished the cytotoxicity of 15dPMJ_2_ [13]. These results demonstrate that ER stress regulates both 15dPMJ_2_-induced DAMP exposure and death and thus, its immunogenicity. Interestingly, the requirement for ER stress in DAMP display and death also dictates whether an agent is classified as a type I or type II ICD inducer [15]. Agents that trigger type I ICD, such as doxorubicin and oxaliplatin, require ER stress for DAMP induction but not for cell death which is mediated by ER stress-independent mechanisms. In contrast, type II agents, including Hyp-PDT, depend on ER stress for both DAMP exposure and cell death [15]. Moreover, for type II ICD inducers including Hyp-PDT, a greater variety and intensity of DAMPs were exposed [9, 16]. Hence, it has been postulated that type II ICD inducers are more efficacious than type I agents [9, 15]. Consistent with this proposal, 15dPMJ_2_ caused a substantial increase in the exposure of different DAMPs including CRT, ATP, HMGB1, Hsp70 and Hsp90. In addition, notably lower concentrations of 15dPMJ_2_ were needed to elicit ICD than the type I inducer, oxaliplatin. This suggests that 15dPMJ_2_ is a tumor-selective, type II ICD inducer.

## MATERIALS AND METHODS

### Antibodies and reagents

15dPMJ_2_ and neutral-15dPMJ_2_ were synthesized as described previously [13]. GSK2606414 was purchased from Millipore Sigma (Burlington, MA, USA). The reagents, 2-mercapthoethanol, propidium iodide and tetradecanoylphorbol acetate (TPA), were purchased from Sigma-Aldrich (St. Louis, MO, USA). GAPDH antibody was obtained from Cell Signaling Technologies (Beverley, MA, USA). Anti-CHOP10 was acquired from Santa Cruz Biotechnology (Santa Cruz, CA, USA). Caspase-Glo^®^ 3/7 assay kit, CellTiter-Glo 2.0 assay kit, and MTS reagent were purchased from Promega Life Sciences (Madison, WI, USA). Anti-calreticulin (Alexa Fluor 647) was from Abcam (Cambridge, MA, USA). HMGB1 ELISA kit was purchased from Novatein Biosciences (Woburn, MA, USA). Recombinant mouse GM-CSF, 7-AAD, PE-Cy7-anti-CD80, APC-Cy7-anti-CD86, PE-Anti-Hsp90 and FITC-Anti-Hsp70 were obtained from BioLegend (San Diego, CA, USA). APC-anti-CD11c and mouse Fc block were purchased from BD Biosciences (San Jose, CA, USA). FITC-anti-MHC class II, anti-CRT and CellTracker Green CMFDA were purchased from Thermo Fisher Scientific (Waltham, MA, USA). IRDye 800CW anti-rabbit and IRDye 680RD anti-mouse secondary antibodies were from LI-COR Biosciences (Lincoln, NE, USA).

### Cell culture

The murine melanoma cell line, B16F10, was purchased from ATCC (Manassas, VA) and cultured in Dulbecco’s minimal essential media (Invitrogen, Carlsbad, CA, USA) containing 10% heat-inactivated fetal bovine serum (FBS), penicillin (100 units/ml) and streptomycin (100 μg/ml). The murine melanocyte cell line, Melan-A, was purchased from the Bennett-Sviderskaya laboratory (Molecular Cell Sciences Research Centre, St. George's, University of London, UK) and cultured in RPMI1640 medium (Thermo Fisher Scientific, Waltham, MA, USA) supplemented with 10% fetal calf serum, penicillin (100 units/ml), streptomycin (100 μg/ml), glutamine (200 μM), and tetradecanoylphorbol acetate (200 nmol/L). The human colon cancer cell line HT29 was purchased from ATCC (Manassas, VA, USA) and cultured in McCoy's 5A medium (Sigma Aldrich, St. Louis, MO, USA) containing 10% heat-inactivated FBS, penicillin (100 units/ml), and streptomycin (100 μg/ml). The non-tumorigenic human keratinocyte cell line, HaCaT, was purchased from Cell Line Service (Eppelheim, Germany). The human squamous carcinoma cell line, A431, was obtained from ATCC (Manassas, VA, USA). Both HaCaT and A431 cells were cultured in Dulbecco's minimal essential media (Invitrogen, Carlsbad, CA, USA) containing 10% heat-inactivated FBS, penicillin (100 units/ml) and streptomycin (100 μg/ml).

### MTS cell viability assay

B16F10 or Melan-A cells were plated in 96-well plates and cultured for 48 hours. Serum-free media containing the listed concentration of different agents was added to the cells for the indicated period of time. MTS reagent (Promega, Madison, WI, USA) was then added to each well and the absorbance at 495 nm was measured according to the manufacturer’s instructions. Absorbance readings were acquired using the Infinite 200 Pro plate reader (Tecan Trading AG, Switzerland).

### Caspase 3/7 activity assay

Cultured cells were plated in white-walled 96-well plates and incubated for 48 hours. Serum-free media containing different concentrations of agents was added to the cells for the indicated time. Caspase-Glo 3/7 reagent (Promega, Madison, WI, USA) was added to each well as directed by the manufacturer. The Caspase-Glo 3/7 kit measures the activity of the executioner caspases 3 and 7 using the luminogenic substrate, Z-DEVD-aminoluciferin. Luminescence was measured using the Infinite 200 Pro plate reader (Tecan Trading AG, Switzerland).

### Detection of apoptosis

Apoptotic cells were detected using Annexin V Alexa Fluor 488 (Thermo Fisher Scientific, Waltham, MA) and the viability dye 7-AAD (BioLegend, San Diego, CA). Briefly, agent-treated B16F10 cells were resuspended in annexin binding buffer (10 mM HEPES, 140 mM NaCl, 2.5 mM CaCl_2_, pH 7.4) containing Annexin V and 7-AAD and then incubated at room temperature for 15 minutes. The samples were analyzed using LSRII flow cytometer (Becton Dickinson, San Jose, CA, USA) and FCS Express V6 software (De Novo Software, Pasadena, CA, USA).

### Extracellular ATP measurement

Cells were cultured for 48 hours and then treated with different agents for the indicated amount of time. ATP levels in the culture medium were quantified using the CellTiter-Glo 2.0 assay kit (Promega, Madison, WI, USA) according to the manufacturer’s instructions. Luciferin is mono-oxygenated by the luciferase enzyme in the presence of ATP producing luminescent signal. Therefore, the CellTiter-Glo 2.0 kit measures the generation of luminescent substrate. The intensity of the luminescent signal is proportional to the amount of ATP that is released into the culture medium by the cells. Luminescence was measured using the Infinite 200 Pro plate reader (Tecan Trading AG, Switzerland).

### Extracellular HMGB1 detection

Cells were cultured for 48 hours and then treated with the appropriate agents for the indicated period of time. The quantity of HMGB1 in the culture media was measured by utilizing the Mouse HMGB1 Sensitive ELISA Kit (Novatein Biosciences, Woburn, MA, USA) according to the manufacturer’s instructions. Absorbance readings were acquired using the Infinite 200 Pro plate reader (Tecan Trading AG, Switzerland).

### Western blot analysis

Western blot analysis was conducted as described previously [35]. Briefly, cells were grown in 100 mm tissue culture dishes for 48 hours and treated with the reagents shown in the text. For vehicle-treated samples, dimethyl sulfoxide (DMSO) was added to serum-free culture medium at a maximum concentration of 0.1% (v/v). After experimentation, the cells were washed twice with ice-cold phosphate buffer saline (PBS). The cells were then lysed by scraping the dishes with 100 μl triton lysis buffer (TLB; 25 mM HEPES, 100 mM NaCl, 1 mM EDTA, 10% (v/v) glycerol, 1% (v/v) Triton X-100) containing protease and phosphatase inhibitors. Protein concentrations were determined by using BCA reagents (Thermo Fisher Scientific, Waltham, MA, USA). Equal concentrations of each sample were loaded onto SDS-PAGE gels and protein bands were transferred to nitrocellulose membranes using semi-dry transfer cells (TRANS-BLOTSD; Bio-Rad Laboratories, Hercules, CA, USA). The membranes were then incubated at room temperature with Odyssey blocking buffer (LI-COR Biosciences, Lincoln, NE, USA) for two hours. Next, the membranes were incubated overnight with anti-CHOP10 (1:500) or anti-GAPDH (1:10,000) primary antibodies and then they were exposed to the appropriate secondary antibody for one hour. All secondary antibodies were used at a dilution of 1:15,000. Protein bands were visualized using Odyssey^®^ CLx digital fluorescence imaging system (LI-COR Biosciences, Lincoln, NE, USA). The band intensities were quantified by using ImageJ software [36].

### Flow cytometric analysis of cell surface CRT, Hsp70, and Hsp90

B16F10 and Melan-A cells were treated with 15dPMJ_2_, oxaliplatin, vehicle (0.1% DMSO) or the cells were left untreated for 2 or 4 hours. The cells were collected, washed twice with FACS Buffer (PBS, 0.5% bovine serum albumin and 0.1% sodium azide) and incubated for 1 hour with Alexa Fluor 647-Anti-calreticulin, PE-Anti-Hsp90, or FITC-Anti-Hsp70 antibodies diluted (1:100) in FACS buffer. The cells were washed twice with FACS buffer before conducting the analysis with the LSR II flow cytometer (BD Biosciences, San Jose, CA, USA). The cell surface expression of CRT, Hsp70 or Hsp90 was quantified using FCS Express V6 software (De Novo Software, Pasadena, CA, USA).

### Extraction of mouse bone marrow-derived dendritic cells (BMDCs)

BMDCs were obtained from the femurs and tibias of 7–9 week-old C57BL/6 mice. The DCs were differentiated for 8 days using RPMI 1640 medium (Thermo Fisher Scientific, Waltham, MA, USA) supplemented with 5% heat-inactivated FBS, L-glutamine (0.03%), sodium pyruvate (0.4 mM), 2-mercapthoethanol (50 μM), mGM-CSF (20 ng/ml), penicillin (100 units/ml) and streptomycin (100 μg/ml).

### Phagocytosis assay

B16F10 and Melan-A cells were labeled with 1 μM CellTracker Green CMFDA (Thermo Fisher Scientific, Waltham, MA, USA) for 30 min. The cells were then treated with 15dPMJ_2_, oxaliplatin or vehicle (0.1% DMSO) for 24 hours. The cells were collected and then co-cultured with BMDCs (generated as described above) in a 1:1 ratio for 2 hours. The co-cultured cells were harvested, incubated with a mouse Fc block, immunostained with APC-anti-CD11c (1:100), and the data was acquired by using the LSR II flow cytometer (BD Biosciences, San Jose, CA, USA). Data analysis was performed using FCS Express V6 software (De Novo Software, Pasadena, CA, USA). BMDCs that phagocytosed CMFDA-labeled dead cell material were identified as CD11c+CMFDA+ double-positive cells.

### Detection of BMDC maturation

B16F10 and Melan-A cells were treated with 15dPMJ_2_, oxaliplatin or vehicle (0.1% DMSO) for 24 hours. The cells were collected and co-cultured in a 10:1 ratio with BMDCs (generated as described above) for 24 hours at 37°C. The co-cultured cells were then collected, washed once in FACS buffer and incubated with mouse Fc block for 10 minutes. The cells were then immunostained with APC-anti-CD11c (1:100), FITC-anti-MHC class II (1:500), PE-Cy7-anti-CD80 (1:100) and APC-Cy7-anti-CD86 (1:100) for 30 minutes. The expression of maturation markers; MHCII, CD86 and CD80 on the surface of CD11c^+^ BMDCs was analyzed using the BD LSR II flow cytometer and FCS Express 6 software.

### Statistical analysis

All data are representative of at least three independent experiments. The data are presented as the mean ± standard error of the mean (SEM). One-way analysis of variance (ANOVA) followed by Tukey’s post-hoc analysis was carried out using GraphPad Prism 5 software (GraphPad Software, San Diego, California).

## SUPPLEMENTARY MATERIALS



## Abbreviations

15dPMJ_2_: 15deoxy, Δ^12,14^ prostamide J_2_
BMDCs: bone marrow-derived dendrtitic cells
CRT: calreticulin
DAMPs: damage-associated molecular patterns
DCs: dendritic cells
ER: endoplasmic reticulum
Hsp: heat shock protein
HMGB1: high-mobility group box 1
ICD: immunogenic cell death

## References

[1] Vinay DS , Ryan EP , Pawelec G , Talib WH , Stagg J , Elkord E , Lichtor T , Decker WK , Whelan RL , Kumara H , Signori E , Honoki K , Georgakilas AG , et al. Immune evasion in cancer: Mechanistic basis and therapeutic strategies. Semin Cancer Biol. 2015; 35:S185–S98. 10.1016/j.semcancer.2015.03.004. 25818339

[2] Zitvogel L , Kroemer G . Targeting PD-1/PD-L1 interactions for cancer immunotherapy. Oncoimmunology. 2012; 1:1223–5. 10.4161/onci.21335. 23243584 PMC3518493

[3] Serrano-Del Valle A , Anel A , Naval J , Marzo I . Immunogenic Cell Death and Immunotherapy of Multiple Myeloma. Front Cell Dev Biol. 2019; 7:50. 10.3389/fcell.2019.00050. 31041312 PMC6476910

[4] Vacchelli E , Galluzzi L , Fridman WH , Galon J , Sautes-Fridman C , Tartour E , Kroemer G . Trial watch: Chemotherapy with immunogenic cell death inducers. Oncoimmunology. 2012; 1:179–88. 10.4161/onci.1.2.19026. 22720239 PMC3376992

[5] Obeid M , Tesniere A , Ghiringhelli F , Fimia GM , Apetoh L , Perfettini JL , Castedo M , Mignot G , Panaretakis T , Casares N , Metivier D , Larochette N , van Endert P , et al. Calreticulin exposure dictates the immunogenicity of cancer cell death. Nat Med. 2007; 13:54–61. 10.1038/nm1523. 17187072

[6] Garg AD , Nowis D , Golab J , Vandenabeele P , Krysko DV , Agostinis P . Immunogenic cell death, DAMPs and anticancer therapeutics: an emerging amalgamation. Biochim Biophys Acta. 2010; 1805:53–71. 10.1016/j.bbcan.2009.08.003. 19720113

[7] Kepp O , Galluzzi L , Martins I , Schlemmer F , Adjemian S , Michaud M , Sukkurwala AQ , Menger L , Zitvogel L , Kroemer G . Molecular determinants of immunogenic cell death elicited by anticancer chemotherapy. Cancer Metastasis Rev. 2011; 30:61–9. 10.1007/s10555-011-9273-4. 21249425

[8] Radogna F , Diederich M . Stress-induced cellular responses in immunogenic cell death: Implications for cancer immunotherapy. Biochem Pharmacol. 2018; 153:12–23. 10.1016/j.bcp.2018.02.006. 29438676

[9] Garg AD , Krysko DV , Verfaillie T , Kaczmarek A , Ferreira GB , Marysael T , Rubio N , Firczuk M , Mathieu C , Roebroek AJ , Annaert W , Golab J , de Witte P , et al. A novel pathway combining calreticulin exposure and ATP secretion in immunogenic cancer cell death. EMBO J. 2012; 31:1062–79. 10.1038/emboj.2011.497. 22252128 PMC3298003

[10] Elhassanny AEM , Soliman E , Marie M , McGuire P , Gul W , ElSohly M , Van Dross R . Heme-Dependent ER Stress Apoptosis: A Mechanism for the Selective Toxicity of the Dihydroartemisinin, NSC735847, in Colorectal Cancer Cells. Front Oncol. 2020; 10:965. 10.3389/fonc.2020.00965. 32626657 PMC7313430

[11] Panaretakis T , Kepp O , Brockmeier U , Tesniere A , Bjorklund AC , Chapman DC , Durchschlag M , Joza N , Pierron G , van Endert P , Yuan J , Zitvogel L , Madeo F , et al. Mechanisms of pre-apoptotic calreticulin exposure in immunogenic cell death. EMBO J. 2009; 28:578–90. 10.1038/emboj.2009.1. 19165151 PMC2657583

[12] Martins I , Kepp O , Schlemmer F , Adjemian S , Tailler M , Shen S , Michaud M , Menger L , Gdoura A , Tajeddine N , Tesniere A , Zitvogel L , Kroemer G . Restoration of the immunogenicity of cisplatin-induced cancer cell death by endoplasmic reticulum stress. Oncogene. 2011; 30:1147–58. 10.1038/onc.2010.500. 21151176

[13] Ladin DA , Soliman E , Escobedo R , Fitzgerald TL , Yang LV , Burns C , Van Dross R . Synthesis and Evaluation of the Novel Prostamide, 15-Deoxy, Delta(12,14)-Prostamide J2, as a Selective Antitumor Therapeutic. Mol Cancer Ther. 2017; 16:838–49. 10.1158/1535-7163.MCT-16-0484. 28292936

[14] Kepp O , Senovilla L , Vitale I , Vacchelli E , Adjemian S , Agostinis P , Apetoh L , Aranda F , Barnaba V , Bloy N , Bracci L , Breckpot K , Brough D , et al. Consensus guidelines for the detection of immunogenic cell death. Oncoimmunology. 2014; 3:e955691. 10.4161/21624011.2014.955691. 25941621 PMC4292729

[15] Krysko DV , Garg AD , Kaczmarek A , Krysko O , Agostinis P , Vandenabeele P . Immunogenic cell death and DAMPs in cancer therapy. Nat Rev Cancer. 2012; 12:860–75. 10.1038/nrc3380. 23151605

[16] Garg AD , Krysko DV , Vandenabeele P , Agostinis P . Hypericin-based photodynamic therapy induces surface exposure of damage-associated molecular patterns like HSP70 and calreticulin. Cancer Immunol Immunother. 2012; 61:215–21. 10.1007/s00262-011-1184-2. 22193987 PMC11029694

[17] Sukkurwala AQ , Adjemian S , Senovilla L , Michaud M , Spaggiari S , Vacchelli E , Baracco EE , Galluzzi L , Zitvogel L , Kepp O , Kroemer G . Screening of novel immunogenic cell death inducers within the NCI Mechanistic Diversity Set. Oncoimmunology. 2014; 3:e28473. 10.4161/onci.28473. 25050214 PMC4063139

[18] Yang Y , Li XJ , Chen Z , Zhu XX , Wang J , Zhang LB , Qiang L , Ma YJ , Li ZY , Guo QL , You QD . Wogonin induced calreticulin/annexin A1 exposure dictates the immunogenicity of cancer cells in a PERK/AKT dependent manner. PLoS One. 2012; 7:e50811. 10.1371/journal.pone.0050811. 23251389 PMC3520942

[19] Wang X , Ji J , Zhang H , Fan Z , Zhang L , Shi L , Zhou F , Chen WR , Wang H , Wang X . Stimulation of dendritic cells by DAMPs in ALA-PDT treated SCC tumor cells. Oncotarget. 2015; 6:44688–702. 10.18632/oncotarget.5975. 26625309 PMC4792585

[20] Zhou J , Wang G , Chen Y , Wang H , Hua Y , Cai Z . Immunogenic cell death in cancer therapy: Present and emerging inducers. J Cell Mol Med. 2019; 23:4854–65. 10.1111/jcmm.14356. 31210425 PMC6653385

[21] Vandenabeele P , Vandecasteele K , Bachert C , Krysko O , Krysko DV . Immunogenic Apoptotic Cell Death and Anticancer Immunity. Adv Exp Med Biol. 2016; 930:133–49. 10.1007/978-3-319-39406-0_6. 27558820

[22] Ma Y , Adjemian S , Mattarollo SR , Yamazaki T , Aymeric L , Yang H , Portela Catani JP , Hannani D , Duret H , Steegh K , Martins I , Schlemmer F , Michaud M , et al. Anticancer chemotherapy-induced intratumoral recruitment and differentiation of antigen-presenting cells. Immunity. 2013; 38:729–41. 10.1016/j.immuni.2013.03.003. 23562161

[23] Zhou H , Mondragon L , Xie W , Mauseth B , Leduc M , Sauvat A , Gomes-da-Silva LC , Forveille S , Iribarren K , Souquere S , Bezu L , Liu P , Zhao L , et al. Oncolysis with DTT-205 and DTT-304 generates immunological memory in cured animals. Cell Death Dis. 2018; 9:1086. 10.1038/s41419-018-1127-3. 30352991 PMC6199251

[24] Fucikova J , Becht E , Iribarren K , Goc J , Remark R , Damotte D , Alifano M , Devi P , Biton J , Germain C , Lupo A , Fridman WH , Dieu-Nosjean MC , et al. Calreticulin Expression in Human Non-Small Cell Lung Cancers Correlates with Increased Accumulation of Antitumor Immune Cells and Favorable Prognosis. Cancer Res. 2016; 76:1746–56. 10.1158/0008-5472.CAN-15-1142. 26842877

[25] Fucikova J , Truxova I , Hensler M , Becht E , Kasikova L , Moserova I , Vosahlikova S , Klouckova J , Church SE , Cremer I , Kepp O , Kroemer G , Galluzzi L , et al. Calreticulin exposure by malignant blasts correlates with robust anticancer immunity and improved clinical outcome in AML patients. Blood. 2016; 128:3113–24. 10.1182/blood-2016-08-731737. 27802968 PMC5201098

[26] Liu P , Zhao L , Loos F , Marty C , Xie W , Martins I , Lachkar S , Qu B , Waeckel-Enee E , Plo I , Vainchenker W , Perez F , Rodriguez D , et al. Immunosuppression by Mutated Calreticulin Released from Malignant Cells. Mol Cell. 2020; 77:748–60.e9. 10.1016/j.molcel.2019.11.004. 31785928

[27] Novitskiy SV , Ryzhov S , Zaynagetdinov R , Goldstein AE , Huang Y , Tikhomirov OY , Blackburn MR , Biaggioni I , Carbone DP , Feoktistov I , Dikov MM . Adenosine receptors in regulation of dendritic cell differentiation and function. Blood. 2008; 112:1822–31. 10.1182/blood-2008-02-136325. 18559975 PMC2518889

[28] Ryzhov S , Novitskiy SV , Goldstein AE , Biktasova A , Blackburn MR , Biaggioni I , Dikov MM , Feoktistov I . Adenosinergic regulation of the expansion and immunosuppressive activity of CD11b+Gr1+ cells. J Immunol. 2011; 187:6120–9. 10.4049/jimmunol.1101225. 22039302 PMC3221925

[29] Ma Y , Adjemian S , Yang H , Catani JP , Hannani D , Martins I , Michaud M , Kepp O , Sukkurwala AQ , Vacchelli E , Galluzzi L , Zitvogel L , Kroemer G . ATP-dependent recruitment, survival and differentiation of dendritic cell precursors in the tumor bed after anticancer chemotherapy. Oncoimmunology. 2013; 2:e24568. 10.4161/onci.24568. 23894718 PMC3716753

[30] Kazama H , Ricci JE , Herndon JM , Hoppe G , Green DR , Ferguson TA . Induction of immunological tolerance by apoptotic cells requires caspase-dependent oxidation of high-mobility group box-1 protein. Immunity. 2008; 29:21–32. 10.1016/j.immuni.2008.05.013. 18631454 PMC2704496

[31] Yang H , Lundback P , Ottosson L , Erlandsson-Harris H , Venereau E , Bianchi ME , Al-Abed Y , Andersson U , Tracey KJ , Antoine DJ . Redox modification of cysteine residues regulates the cytokine activity of high mobility group box-1 (HMGB1). Mol Med. 2012; 18:250–9. 10.2119/molmed.2011.00389. 22105604 PMC3324950

[32] Apetoh L , Ghiringhelli F , Tesniere A , Obeid M , Ortiz C , Criollo A , Mignot G , Maiuri MC , Ullrich E , Saulnier P , Yang H , Amigorena S , Ryffel B , et al. Toll-like receptor 4-dependent contribution of the immune system to anticancer chemotherapy and radiotherapy. Nat Med. 2007; 13:1050–9. 10.1038/nm1622. 17704786

[33] Ramos-Casals M , Brahmer JR , Callahan MK , Flores-Chavez A , Keegan N , Khamashta MA , Lambotte O , Mariette X , Prat A , Suarez-Almazor ME . Immune-related adverse events of checkpoint inhibitors. Nat Rev Dis Primers. 2020; 6:38. 10.1038/s41572-020-0160-6. 32382051 PMC9728094

[34] Wei SC , Duffy CR , Allison JP . Fundamental Mechanisms of Immune Checkpoint Blockade Therapy. Cancer Discov. 2018; 8:1069–86. 10.1158/2159-8290.CD-18-0367. 30115704

[35] Elhassanny AEM , Ladin DA , Soliman E , Albassam H , Morris A , Kobet R , Thayne K , Burns C , Danell AS , Van Dross R . Prostaglandin D2-ethanolamide induces skin cancer apoptosis by suppressing the activity of cellular antioxidants. Prostaglandins Other Lipid Mediat. 2019; 142:9–23. 10.1016/j.prostaglandins.2019.03.001. 30858059

[36] Rueden CT , Schindelin J , Hiner MC , DeZonia BE , Walter AE , Arena ET , Eliceiri KW . ImageJ2: ImageJ for the next generation of scientific image data. BMC Bioinformatics. 2017; 18:529. 10.1186/s12859-017-1934-z. 29187165 PMC5708080

