# Supplementary material for: Damage-associated molecular pattern (DAMP) activation in melanoma: investigation of the immunogenic activity of 15-deoxy, Δ12,14 prostamide J2
Source: Oncotarget. 2020 Dec 29;11(52):4788–802. doi: 10.18632/oncotarget.27856 (PMC7779254; doi:10.18632/oncotarget.27856)
Supplement: Supplementary file 1 [file oncotarget-11-4788-s001.pdf]

Damage-associated molecular pattern (DAMP) activation in melanoma: investigation of the immunogenic activity of 15-deoxy, $\Delta^{12,14}$ prostamide J_2

SUPPLEMENTARY MATERIALS

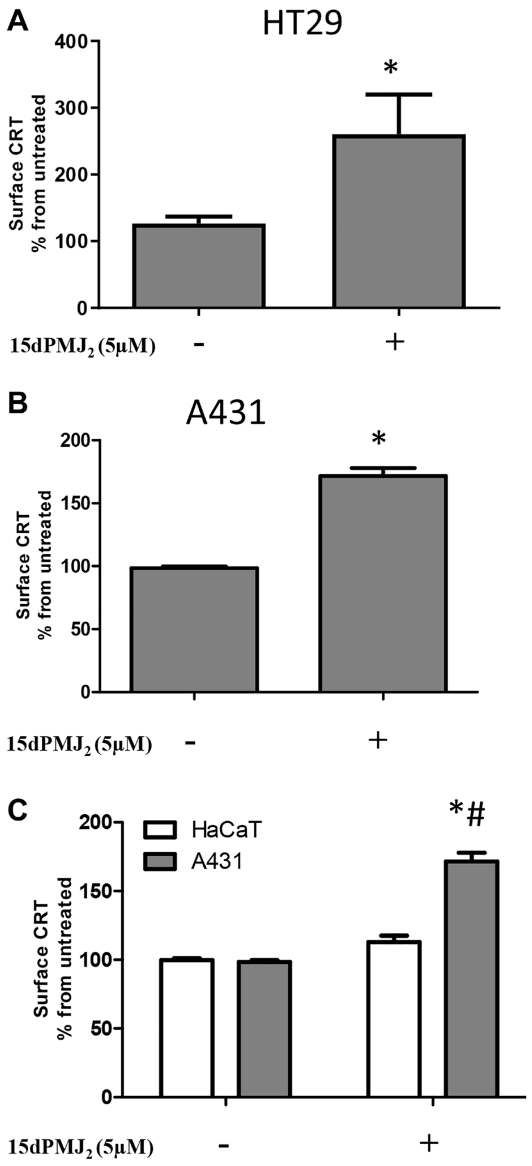

Supplementary Figure 1: 15dPMJ$_2$ increases cell surface calreticulin (CRT) expression in both colorectal and non-melanoma skin cancer cells. (**A**) Colorectal cancer (HT29) cells or (**B** and **C**) tumorigenic (A431) and non-tumorigenic (HaCaT) keratinocytes were treated with vehicle (0.1% DMSO), 5 µM 15dPMJ$_2$, or the cells were left untreated. The cell surface expression of CRT was measured by conducting flow cytometric analysis after treatment for 2 hours. The data in A and B were analyzed using Student's *t*-test. The data in C were analyzed using one-way ANOVA followed by Tukey's multiple comparison test and are represented as the mean ± SEM of three independent experiments. $^{*}p < 0.05$, sample compared to vehicle treated cells; $^{\#}p < 0.05$, sample compared to HaCaT cells.

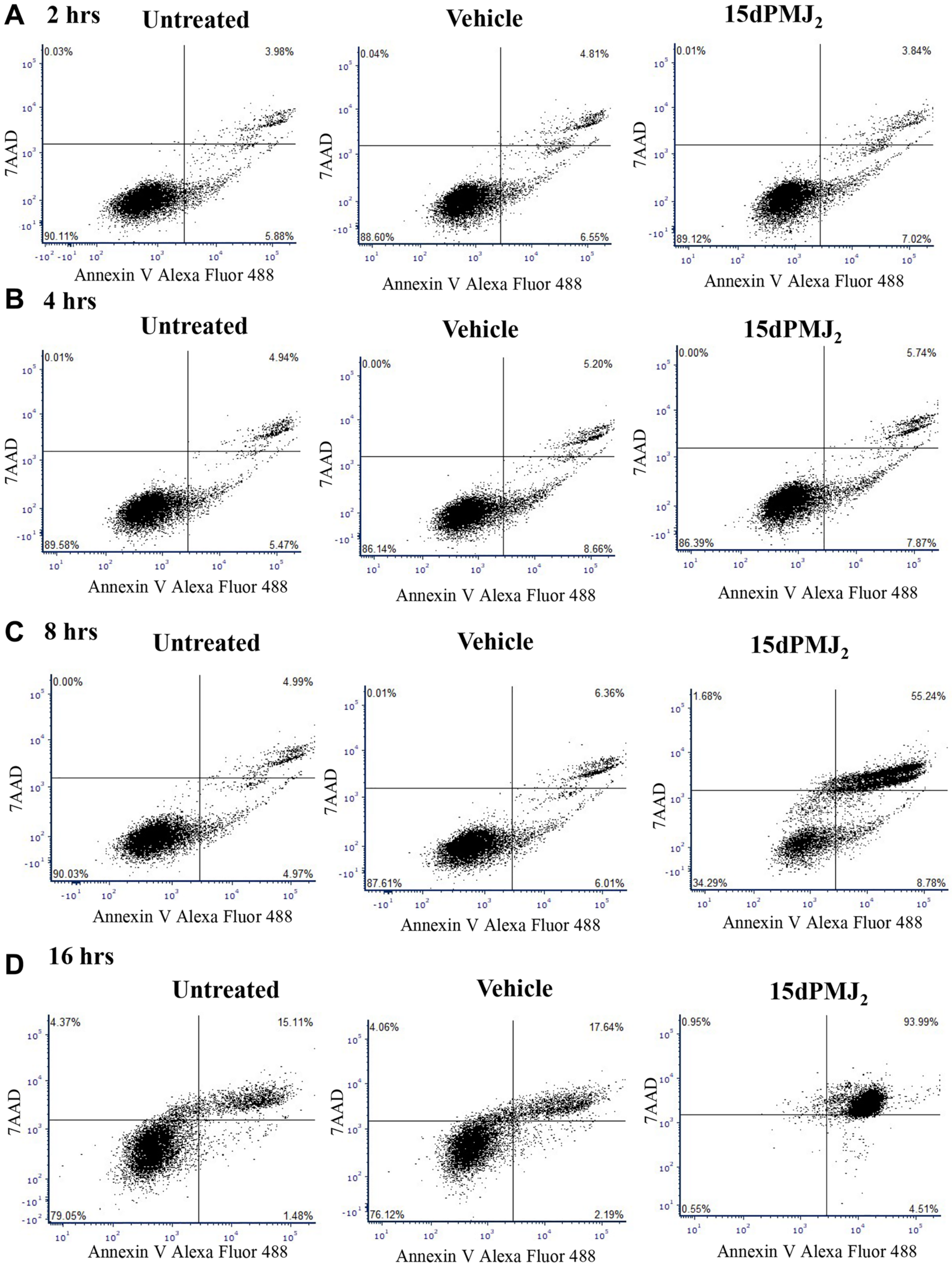

Supplementary Figure 2: Time course of 15dPMJ$_2$-induced apoptosis in B16F10 cells. B16F10 cells were treated with vehicle (0.1% DMSO), 5 μM 15dPMJ$_2$ or the cells were left untreated for (**A**) 2 hours, (**B**) 4 hours, (**C**) 8 hours or (**D**) 16 hours. Cells were stained with Annexin V Alexa Fluor 488 and 7-AAD. Samples were analyzed using LSRII flow cytometer and FCS Express 6 software.
